# Influenza Vaccination Strategies in Healthcare Workers: A Cohort Study (2018–2021) in an Italian University Hospital

**DOI:** 10.3390/vaccines9090971

**Published:** 2021-08-30

**Authors:** Marco Dettori, Antonella Arghittu, Giovanna Deiana, Antonio Azara, Maria Dolores Masia, Alessandra Palmieri, Antonio Lorenzo Spano, Antonello Serra, Paolo Castiglia

**Affiliations:** 1 Department of Medical, Surgical and Experimental Sciences, University Hospital of Sassari, 07100 Sassari, Italy; madettori@uniss.it (M.D.); azara@uniss.it (A.A.); luca@uniss.it (A.P.); antonio.spano@aousassari.it (A.L.S.); antonello.serra@aousassari.it (A.S.); castigli@uniss.it (P.C.); 2 Department of Medical, Surgical and Experimental Sciences, University of Sassari, 07100 Sassari, Italy; mdmasia@uniss.it; 3 Department of Biomedical Sciences, University of Sassari, 07100 Sassari, Italy; giovanna.deiana90@gmail.com

**Keywords:** flu vaccination, healthcare workers, vaccination coverage, on-site offer, vaccination strategies

## Abstract

Vaccinating healthcare workers (HCWs) is the most effective intervention for preventing nosocomial influenza infection. However, influenza vaccination coverage (VC%) among HCWs remains low. The aim of the study was to analyse the trend of VC% among healthcare workers in an Italian hospital through a three-year vaccination project covering three influenza seasons (2018–2019, 2019–2020, and 2020–2021). A gap analysis was performed at the case base (2018–2019), on-site vaccination was trialled in the 2019–2020 season, and an integrated vaccination offer (on-site vaccination and the classic offer at a vaccination clinic) was implemented for the 2020–2021 season. For each unit of vaccinated HCWs, the following variables were recorded: main demographic details, area of affiliation (medical/surgical/services), and professional category. Logistic regression analyses were performed to assess the relationship between outcome (undergoing vaccination in 2020–2021) and other variables related to the healthcare workers’ characteristics. In the three seasons, VC% values of 13.2%. 27.7%, and 58.9% were recorded, respectively (*p* < 0.005). The highest VC% was recorded among physicians (94.93%), in the medical area (63.27%), and males (62.59%) and in general among the youngest HCWs. Comparison of the coverage values recorded in the three seasons showed that in the last season considered (2020–2021) about 80% of health workers preferred to be vaccinated in the workplace instead of using the standard vaccination delivery method (invitation to attend the vaccination clinic). Our study suggests that the integrated vaccination offer may lead to an increase in VC% among HCWs compared to the classical offer modalities.

## 1. Introduction

Seasonal influenza is a public health problem and a major source of direct and indirect costs arising from the case management and complications of the disease. Indeed, influenza viruses have a major epidemiological, social, and economic impact on industrialised countries [1,2,3]. Currently, according to the World Health Organisation (WHO), the number of influenza-related deaths worldwide ranges from 250,000 to 650,000, while according to the European Centre for Disease Prevention and Control (ECDC), between 4 and 50 million people contract symptomatic influenza in Europe every year, resulting in 15,000 to 70,000 deaths [1,4,5,6]. In Italy alone, 5 to 8 million people are affected every year, with a case fatality rate of 8000 deaths/year [6,7,8]. Ninety percent of deaths occur in subjects over 65 years of age, in particular among those with co-morbidities [7].

The most effective preventive strategy available to reduce the burden of the disease is vaccination [9,10,11,12,13]. Adherence to flu vaccination is especially important in the current pandemic, as the initial symptoms of COVID-19 are very similar to those of influenza and, therefore, the concomitance of seasonal influenza and SARS-CoV-2 infection would place an additional burden on the health service, with increasing difficulties for physicians to reach diagnosis [13,14]. In this regard, the Strategic Advisory Group of Experts on Immunization (SAGE) of the World Health Organization (WHO) recommends offering influenza vaccination with priority given to healthcare workers, adults ≥ 65 years, pregnant women, individuals with underlying health conditions, and children aged 6–59 months [11,12,13]. In Italy, the same priority groups are recommended yearly by the Ministry of Health and the Regions [15].

Among the indicated cohorts, healthcare workers (HCWs) are particularly at risk of contracting influenza (clinical and subclinical) and of transmitting the infection to patients whose underlying conditions increase their risk of complications [8,16].

Several studies have investigated the causes of low compliance by healthcare professionals. These have found that the main determinants of hesitation are: (i) inadequate awareness campaigns; (ii) altered risk perception; (iii) insufficient health education on the efficacy of the influenza vaccine and/or possible adverse reactions; (iv) lack of access to vaccination facilities; (v) socio-demographic variables. In addition, several authors state that one of the main determinants of the low uptake of the flu vaccine for healthcare workers is a lack of time to attend the vaccination clinic [8,17,18,19,20].

Among the policies recommended by international public health organisations to improve vaccination coverage among healthcare workers, the Centre for Disease Control and Prevention (CDC) cites on-site influenza vaccination as a proven and cost-effective strategy that increases productivity, reduces sick leave, and improves vaccination adherence among HCWs [21,22,23].

Bearing in mind the importance of flu vaccination for HCWs and aiming to boost adherence, in Italy, the regulation of vaccination for this category is further provided for by Legislative Decree 9 April 2008 no. 81. This decree recommends actively offering the anti-influenza vaccine to HCWs annually during the flu season, from October to December [15,24,25]. Nevertheless, flu vaccination coverage among HCWs remains low, as it is in other countries [7,8,17,26].

Based on these premises, the present study aims to investigate influenza vaccination coverage among HCWs employed by an Italian University Hospital, comparing the effect of the on-site vaccination strategy with the results from previous flu seasons during which the classic delivery model (invitation to attend the vaccination clinic) was used.

## 2. Materials and Methods

### 2.1. Study Setting

The present study did not require ethical approval for its observational design according to the Italian law (Gazzetta Ufficiale no. 76 dated 31 March 2008).

The vaccination campaign was aimed at employees (3044 employees as of 1 December 2020) of the University Hospital of Sassari, Italy (AOU-SS), of whom 2119 were female and 925 male).

The AOU-SS is the main hospital in the Italian region of Sardinia in terms of the number and diversity of its professional and technological resources, and carries out multi-specialist care, teaching, and research activities for the entire northern territory of Sardinia. The organisational structure of the hospital is set out in the company act (art. 3 paragraph 1 bis of Legislative Decree no. 502/92 and subsequent amendments), which identifies a total of 77 operational units. Based on the type of activity carried out, these units are grouped into macro-areas: 29 medical areas; 18 surgical areas; 30 services/other. Its professional personnel are numerically distributed as shown in Table 1.

The mean age of the staff is 47.64 years with a standard deviation of ±10.80 and the most represented age group is 50–60 with 1013 HCWs, of which 733 are females and 280 are males (Table 2). Distribution by macro-area, age group, and gender of AOU-SS HCWs is shown in Table 3.

### 2.2. Project Planning

As regards the flu vaccination offer, until the 2018–2019 season AOU-SS guaranteed the provision of the vaccination service at a single dedicated vaccination clinic, which the HCWs voluntarily attended to receive the flu jab. This standard practice made flu vaccination available to staff between October of each year and January of the following year and made it possible to reach a vaccination coverage that fluctuated between 13% and 15% of the company population.

The flu vaccination campaign for the 2020–2021 season represents the final phase of a three-year vaccination communication project that included the 2018–2019, 2019–2020, and 2020–2021 seasons. This project involved the implementation of activities divided into 4 distinct phases as described in Table 4.

#### 2.2.1. Flu Vaccination Offer in the 2018–2019 and 2019–2020 Seasons

In particular, a cognitive survey of the company’s population was conducted during the 2018–2019 season.

In order to assess the attitudes, behaviours and knowledge of AOU-SS staff regarding influenza vaccination, an anonymous questionnaire was developed on the EUSurvey digital platform. The questionnaire was administered by sending a URL code via email to employees in the period between November 2018 and March 2019 [8]. From the results of the survey, the authors describe difficulty in accessing vaccination as the main determinant of vaccination hesitation among HCWs.

In light of this variable, new vaccination delivery strategies were devised to be implemented in subsequent influenza seasons.

In addition to routine vaccination activities, these supply strategies included the implementation of a pilot flu vaccination programme administered directly on the ward (catch-up) by a team of specialists who vaccinated those HCWs who requested the vaccine via booking, on-site.

#### 2.2.2. Flu Vaccination Offer in the 2020–2021 Season

The on-site vaccination strategy tested in the previous season was reproposed for the 2020–2021 season with a more articulated organisation structured around the needs of the operational units belonging to the medical, surgical, and service areas.

In particular, care departments with nursing activities (thus, able to self-administer the vaccine) were given the possibility of vaccinating staff directly in their own department, while for operational units and external companies who required medical or nursing staff to administer the jab, vaccination was offered at ad hoc vaccination clinics.

The mode of vaccine administration and venues implemented for the 2020–2021 influenza season are described in Table 5.

The directors of each operational unit received a specific email explaining the vaccination strategies. The same information was also communicated on the AOU-SS website. In the weeks preceding the vaccination campaign, specific posters were displayed in each operational unit, and brochures on the subject of influenza and the importance of flu vaccination were distributed.

Specific forms were drawn up and sent to all the AOU-SS units and to all the managers of external services (maintenance companies, cleaning companies, etc.).

These forms included: (i) a dose request form; (ii) a medical history form to be filled in at the time of vaccination; (iii) an information sheet for the vaccinator and the vaccinee. After completion of these forms, they were then returned to the Hospital Hygiene and Infection Control Unit, which, in collaboration with the Medical Directorate Unit and depending on the availability of vaccines, arranged the scheduling of vaccination deliveries for self-vaccinating wards (with nursing and/or outpatient activities) and vaccination sessions for wards (without nursing and/or outpatient activities) which were unable to self-administer.

As confirmation of vaccination, a form was issued containing the vaccinee’s medical history, consent to vaccination and the batch of vaccine administered.

For each unit of vaccinated health personnel, the following variables were recorded: main demographic details, area of affiliation (medical/surgical/services), and professional category.

Data collection and informed consent were carried out by the staff of the Hospital Hygiene and Infection Control Unit.

For the 2020–2021 season, vaccinated HCWs received one dose of inactivated tetravalent split vaccine, administered intramuscularly into the deltoid; these individuals underwent a two-week follow-up to assess any adverse effects and subsequently reported to the pharmacovigilance system.

### 2.3. Statistical Analysis

Data were entered on Excel (Microsoft Office, Microsoft Corporation, Redmond, WA, USA) and analysed using the STATA software 16 (StatCorp., Austin, TX, USA) and MedCalc (MedCalc Software Ltd., Ostend, Belgium). Continuous variables were expressed as the mean ± standard deviation and range, categorical variables as proportions. For the calculation of the vaccination coverage (VC%), the number of vaccinated health workers was used as a numerator and the number of employees of AOU-SS as of 31 December 2020 (in each OU) as denoter.

Differences among quantitative variables and frequencies were tested through the Student’s t-test and Χ^2^ test, respectively. Differences among proportions were tested with the z test. Linear trend in proportions was tested too. A two-tailed *p*-value of less than 0.05 was considered statistically significant.

Univariate and multivariate logistic regression analyses were performed, and Odds Ratios were used to assess the relationship between the outcome (being vaccinated in 2020–2021) and the following variables related to personal and health workers’ characteristics: age, gender, profession, and area of activities. The outcome was established by attributing a value of 1 if the participant underwent vaccination, and a value of 0 otherwise. A two-tailed *p*-value of less than 0.05 was considered statistically significant.

## 3. Results

### 3.1. Flu Vaccination Coverage in the University Hospital

**Figure 1:**
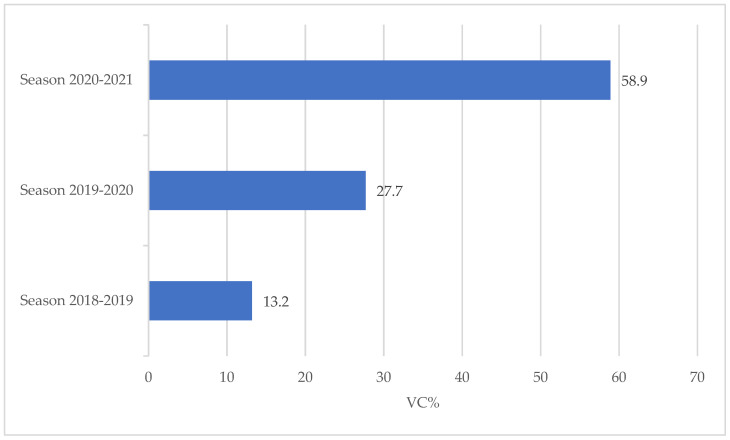
Recorded influenza vaccination coverage (VC%) among AOU-SS healthcare workers in seasons 2018–2019, 2019–2020, and 2020–2021.

Following the first two seasons of the vaccination campaign, the situation regarding the vaccination coverage recorded (VC%) was as follows: in the 2018–2019 season through the ordinary vaccination activities (invitation to attend the vaccination clinic) VC% of 13.20% were recorded among the AOU-SS employees, while in the 2019–2020 season, to the 400 vaccinations administered through the ordinary vaccination activities, an additional 229 ward (on-site) administrations were added for a total of 629 HCWs vaccinated (VC% = 27.70%) (Figure 1).

For the 2020–2021 season, through the combined vaccination offer methods (ordinary activities and ward-based vaccination), 1793 HCWs out of a population of 3044 employees underwent influenza vaccination (VC% = 58.90%).

The increase in coverage in the three seasons under consideration is statistically significant (linear trend; *p* < 0.05).

### 3.2. Results in 2020–2021 Flu Season

In the 2020–2021 season, of the vaccinated HCWs’ cohort, 67.71% were females and 32.29% were males (*p* < 0.05). The age group in which the highest adherence to influenza vaccination was found was 20–30 (VC% of 75.14) (Table 6), with a significant difference between age groups (*p* < 0.05). The mean age of the vaccinated healthcare workers was 46.74 with a standard deviation ± 11.25. The distribution of VC% of HCWs by gender and age group is shown in Table 6.

The vaccination coverage recorded among the HCWs operating in the different areas, distributed by gender and age groups, is shown in Table 7.

In particular, the results show that some statistically significant differences between gender were observed at different age group and in different areas (Table 7).

**Figure 2:**
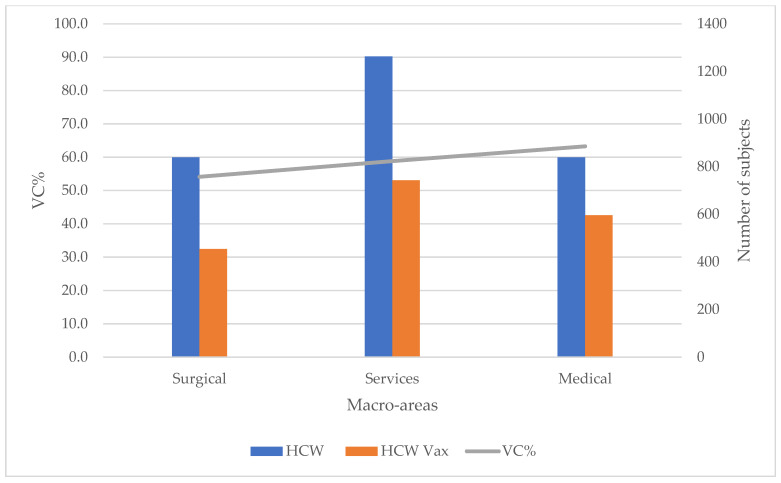
Vaccination coverage (VC%) in the 2020–2021 season of AOU-SS health personnel (HCWs) by macro-area. Vax = vaccinated.

Overall, in the various macro-areas, the recorded vaccination coverage showed values of 54.11% in the surgical area, 58.83% in the services area and 63.27% in the medical area (Figure 2), *p* < 0.05. As far as gender is concerned, a statistically significant difference in VC% was found only in the Service area (*p* < 0.05).

Regarding vaccination adherence among the different professional categories working at AOU-SS, the vaccination coverage distributed by age and sex is shown in Table 8.

In particular, the results show that there is a statistically significant difference between male and female doctors (*p* = 0.0005), whereas there are no significant differences between females and males in the nursing staff and the other HCW categories. On the other hand, overall, males show higher VC% than females (*p* = 0.0063). This is a clear example of Simpson’s paradox due to the different distribution between gender within the categories.

Regarding the relationships between flu vaccination, demographic variables (sex, age), and occupational variables (category and area of affiliation), the regression analysis reported the results shown in Table 9.

Statistically significant variables from the univariate analysis in relation to the outcome (undergoing vaccination in the 2020–2021 flu season) were selected and included in a multivariate logistic regression model. Specifically, AOU-SS HCWs showed a higher propensity for influenza vaccination if: (i) male (Odds Ratio, OR (Confidence Interval, CI95%) = 1.2 (1.0–1.5); *p* = 0.013); (ii) young in age (OR (CI95%) = 1.1 (1.0–1.2); *p* = 0.004); (iii) from the medical staff (OR (CI95%) = 2.3 (2.0–2.5); *p* = 0.000); (iv) from the medical area (OR (CI95%) = 1.5 (1.3–1.8); *p* = 0.000).

Regarding vaccine safety, no serious and/or long-term adverse reactions were observed during the 2 weeks’ follow-up. The most common reactions reported were pain at the injection site and rarely (<1/100) mild fever (<38 °C). All adverse events resolved without sequelae.

## 4. Discussion

This study aimed to investigate the flu vaccination coverage of healthcare workers employed by AOU-SS as an indicator of compliance with this vaccination. The comparison of the coverage values recorded in the seasons 2018–2019 (VC% = 13.20%), 2019–2020 (VC% = 27.70%), and 2020–2021 (VC% = 58.90%) showed that in the last season under consideration (2020–2021) about 80% of HCWs preferred to be vaccinated in the workplace instead of using the standard method of vaccination provision (invitation to attend the vaccination clinic).

Although the vaccination coverage reached in the last year is still unsatisfactory compared to the national minimum values indicated by the Ministry of Health (VC% = 75%) [15,25], our study suggests that the on-site vaccination offer appears to be able to increase vaccination coverage and lead to an improvement in the compliance of health workers. In fact, the new vaccination strategies, which included both routine and on-site vaccination, made it possible to achieve more than three times the coverage values of previous seasons. This percentage was the highest in the years observed and, despite the absence of official statistics on the subject, it was higher than the vaccination coverage reported by the most recent national and European observations [27,28,29,30].

Furthermore, it is interesting to consider that the Ministerial Circular “Influenza prevention and control: recommendations for the 2020–2021 season” recommended bringing forward flu vaccination campaigns from the beginning of October 2020, offering vaccination to eligible individuals at any time during the season [15]. This effectively extended the time available to AOU-SS to reach hospital employees, further influencing the increase in coverage.

Another fact worth bearing in mind is that, given the lengthy duration of the vaccination campaign, the vaccination coverage recorded at AOU-SS for the 2020–2021 season is to be considered as conservative and therefore the authors cannot provide information on the compliance of staff who did not undergo flu vaccination in the hospital. In fact, since vaccines were procured in different tranches over a period between October 2020 and January 2021, it was not possible to assess whether HCWs who were not immunised in the hospital were vaccinated by their general practitioner or if they purchased the vaccine from the pharmacy.

In addition, with regard to the characteristics of the personnel vaccinated in the hospital, our study reports that some professional categories, such as nurses and older HCWs, were less inclined to be vaccinated against influenza, while doctors (especially those in the medical area) were more compliant.

This is particularly important in the current pandemic in which the protection of a subgroup at high risk of contracting influenza, such as older HCWs, is particularly important: older nurses and doctors, who are therefore more experienced due to their professional expertise, are particularly valuable in healthcare emergencies [31,32,33]. Therefore, flu vaccination takes on even greater importance as preventing the disease reduces not only the sequelae associated with it but also absence from work due to illness [7,8,9,10,30,33].

Our results confirm what has been observed by other authors who report low compliance with flu vaccination by older nurses in the surgical area [8,34,35,36]. This may be due to the fact that, in the past, the training of elderly HCWs (especially nurses) was mainly focused on the care of the patient rather than on the prevention of the disease, while in the present-day training of university students, great importance is given to prevention including prophylactic vaccination, with a consequent greater awareness of the issue.

Moreover, it is well known that influenza is often perceived by health professionals as an exaggerated or inapparent risk, compared to the actual incidence of the disease [7,8,13,37,38,39]. The altered perception of the health risk, in this context, has a significant impact on the decision to undergo vaccination and, as such, understanding the factors that influence the perceived risk becomes fundamental to be able to promote a true perception and encourage adequate adherence to preventive measures [37,38,39,40,41,42].

This aspect had previously been highlighted in a survey carried out by the authors in the 2018–2019 season, in which useful indications emerged on the cognitive limits and misperception of the risks associated with influenza, aspects that, together with the declared difficulty of access to the vaccination service, strongly conditioned HCWs’ adherence to vaccination [8]. In view of this, the three-year vaccination project implemented by the OU of Hospital Hygiene and Infection Control, using a strategy based on: (i) the ordinary supply of vaccinations (a single dedicated vaccination clinic) expanded with the setting up of two other vaccination clinics; (ii) vaccination on the ward with self-administration methods; (iii) health education interventions in the field, training, and information; (iv) a communication campaign structured on the needs of the target, has made it possible to achieve encouraging vaccination coverage values.

This gives cause for reflection on the importance of capillarisation of the vaccination offer as a tool to be used in the near future in order to reach HCWs more easily and increase vaccination compliance. Indeed, the on-site offer could be used not only for the administration of the vaccine in light of the staff’s working requirements but could also be a useful opportunity to educate HCWs on the significance of preventive prophylaxis and on the health risks for the individual and patients associated with influenza [43]. This evidence is also confirmed in the literature by several studies describing the variables above as key determinants of vaccination compliance in HCWs [8,43,44,45].

Influenza vaccination among HCWs has been an intensely investigated topic in Italy in recent years, in particular with the aim to evaluate the campaigns’ effectiveness in increasing vaccination coverage [45,46,47]. During the COVID-19 pandemic, the effectiveness of these measures has become particularly important as the influenza vaccine is useful to distinguish between symptoms of influenza and those related to COVID-19 and enables the prevention of outbreaks in hospitals and disruption of health services due to HCWs requiring sick leave [48,49,50]. Furthermore, co-circulation of SARS-CoV-2 and influenza virus during the 2020–2021 season may have caused an increased perception of the importance of flu vaccination among health professionals, leading to greater adherence to vaccination. In support of this interpretation of the survey results, numerous studies state that the perceived level of a health threat is a strong predictor of people’s intention to engage in preventative behaviours, including influenza vaccination [39,40,45]. Therefore, the emergence of COVID-19, in relation to the flu vaccination campaign, may have a significant impact especially in the coming years, involving greater adherence to flu vaccination but also, should it be necessary to repeat vaccination against SARS-CoV-2 annually, timely planning of the two vaccination campaigns and more generally of the vaccination facilities in order to have sufficient and adequately trained personnel.

One important lesson learned from our three-year experience observing the behaviour of HCWs regarding adherence to influenza vaccination in multiple flu seasons is that increasing vaccination coverage among HCWs is not always guaranteed and is often a difficult goal to achieve. In fact, it depends on the synergy of numerous variables linked not only to the availability and provision of the vaccine but also to the presence of adequate and experienced human resources, health education and the promotion of well-structured communication campaigns [8,43,44,45,51,52,53].

In this regard, the flu vaccination campaign promoted at AOU-SS through the use of different communication approaches may have had a positive influence on bringing healthcare personnel closer to the practice of vaccination. These activities included: posting explanatory leaflets and posters in each hospital ward, distributing information material, gaining the endorsement of the Provincial Command of the Fire Brigade who collaborated on the creation of a promotional advert, using social media, and conveying correct communication through websites dedicated to vaccination [52].

In the future, therefore, it would be appropriate to repeat the on-site vaccination strategy, extending the offer to as many wards as possible and trying to involve mainly the professional groups who have the most contact with patients.

## 5. Conclusions

Healthcare professionals have a vital responsibility to their patients to ensure that those in need of medical care are assisted. However, vaccination coverage among health workers continues to be unsatisfactory and this data is also confirmed among health workers in the Italian hospital studied, in which the vaccination coverage recorded in recent years has always had values below 75%, albeit with some improvement.

It is therefore essential to continue to implement strategies to increase vaccination compliance and increase accountability to their patients, as required by national and international recommendations. For these reasons, there is an urgent need for studies aimed at implementing the best strategies to increase vaccination coverage in this cohort of professionals. This would also help policymakers and stakeholders to define specific initiatives and programmes in order to maximise the effects of flu vaccination programmes.

Further efforts are also needed to increase flu vaccination coverage rates among HCWs. This is a priority for public health and can be achieved through well-designed long-term intervention programmes that include a variety of coordinated management and organisational elements (e.g., vaccination offered in hospitals to staff and patients on discharge).

## Figures and Tables

**Table 1:** Distribution of AOU-SS healthcare workers (HCWs) by professional category and gender (F = females; M = males).

Professional Category	No. HCWs	%	F	% F	M	% M
Medical staff	572	18.79	298	9.79	274	9.00
Nursing staff	1144	37.58	951	31.24	193	6.34
Other healthcare staff	1328	43.63	870	28.6	458	15.03

**Table 2:** Distribution of AOU-SS health workers (HCWs) by gender (F = females; M = males) and age group.

Age Group	No. HCW	%HCW	No. HCW F	%HCW F	No. HCW M	%HCW M
20–30	181	5.95	126	5.95	55	5.95
30–40	584	19.19	398	18.78	186	20.11
40–50	808	26.54	579	27.32	229	24.76
50–60	1013	33.28	733	34.59	280	30.27
>60	458	15.05	283	13.36	175	18.92
Total	3044	100.00	2119	100.00	925	100.00

**Table 3:** Distribution by macro-area, age group, and gender (F = females; M = males) of AOU-SS healthcare workers (HCWs).

Macro-Area	Age Group	No.	F	M
Surgical Area	20–30	44	30	14
	30–40	142	92	50
	40–50	237	165	72
	50–60	289	215	74
	>60	127	82	45
	Total	839	584	255
Services Area	20–30	80	56	24
	30–40	246	161	85
	40–50	312	209	103
	50–60	415	286	129
	>60	210	121	89
	Total	1263	833	430
Medical Area	20–30	57	40	17
	30–40	196	145	51
	40–50	259	205	54
	50–60	309	232	77
	>60	121	80	41
	Total	942	702	240

**Table 4:** Phases and activities of the flu vaccination campaign.

Activites	Actions
Gap Analysis	Planning the communication campaign and target analysis
	Administering an ad hoc questionnaire to AOU-SS employees Analysing the results
2. Accessibility to vaccination service	Studying the best strategies for providing flu vaccination
	Implementing a pilot on-site flu vaccination project by an ad hoc team of specialists
	Providing flu vaccination directly on the ward by staff or in ad hoc vaccination clinics to supplement existing vaccination procedures (invitation to attend the company’s only vaccination clinic).
3. Health education and continuous training	Organising focus groups and awareness-raising among the HCWs
	Distributing promotional material (posters, leaflets, pins, t-shirts, caps, multi-year calendars)
4. Promoting flu vaccination in hospitals	Involving Dinamo Banco di Sardegna basketball club to endorse the project in the 2019–2020 season
	Involving the Provincial Command of the Sassari Fire Brigade to endorse the project in the 2020–2021 season
	Communicating widely online (www.vaccinarsisnardegna.org (accessed on 24 June 2021)) and social media

**Table 5:** Mode of flu vaccine administration.

Operational Units	Flu Vaccine Administration/Vaccine Provision
Wards with nursing activities	On-site (i.e., employees of each OU were vaccinated through self-administered methods and timing)
Non-medical wards	Vaccination clinic set up in the Hospital Hygiene and Infection Control Unit Vaccination clinic set up at the Medical Directorate Unit
External companies (e.g., maintenance companies, cleaning companies, waste disposal companies)	Vaccination clinic set up in the Occupational Medicine Unit

**Table 6:** Vaccination coverage (VC%) of AOU-SS healthcare workers (HCWs) undergoing influenza vaccination in 2020–2021 season distributed by age groups and gender (F = females; M = males).

Age Group	VC% HCWs	VC% F	VC% M
20–30	75.14	48.62	87.27
30–40	64.73	42.12	70.97
40–50	57.30	40.47	59.39
50–60	54.79	38.89	57.50
>60	56.99	34.72	58.29

**Table 7:** Distribution of vaccination coverage (VC%) by marco-area of affiliation, age group, and gender (F = females; M = males) of healthcare workers (HCWs) who are receiving the influenza vaccination.

Macro-Area	Age Group	VC% HCWs	VC% F	VC% M	*p*-Value
Surgical Area	20–30	84.09	76.67	100.00	0.0487
	30–40	59.86	54.35	70.00	0.0692
	40–50	54.96	54.55	55.56	0.8857
	50–60	49.13	49.77	47.30	0.7139
	>60	47.24	45.12	51.11	0.5178
	Total	54.71	52.57	57.65	0.1744
Services Area	20–30	65.00	60.71	75.00	0.2195
	30–40	65.45	65.22	65.88	0.9176
	40–50	56.73	52.15	66.02	0.0201
	50–60	54.70	51.40	62.02	0.0443
	>60	60.00	57.85	62.92	0.4586
	Total	58.83	55.82	64.65	0.0025
Medical Area	20–30	82.46	77.50	94.12	0.1312
	30–40	67.35	62.76	80.39	0.0209
	40–50	60.23	62.44	51.85	0.2828
	50–60	60.19	60.34	59.74	0.9395
	>60	61.98	65.00	56.10	0.3398
	Total	63.27	62.96	64.17	0.7371

**Table 8:** Distribution by gender (F = females; M = males; Vax = vaccinated) of recorded vaccination coverage (VC%) among the different professional categories working at AOU-SS.

Professional Categories	F Vax	VC%	M Vax	VC%	*p*-Value
Medical staff	292	97.99	251	91.61	0.0005
Nursing staff	479	50.37	90	46.63	0.3434
Other healthcare workers	443	50.92	238	51.97	0.7159
Total	1214	57.29	579	62.59	0.0063

**Table 9:** Univariate and multivariate analysis. OR = Odds Ratio; CI = Confidence Interval.

Variables	Answers	Univariate Analysis		Multivariate Analysis	
General Information	(Yes/No)	OR (95% CI)	*p*-Value	OR (95% CI)	*p*-Value
Gender (reference = female)	Male	1.2 (1.1–1.5)	0.006	1.2 (1.0–1.5)	0.013
2. Age group (reference = >60)	20–30	1.2 (1.1–1.3)	0.000	1.1 (1.0–1.2)	0.004
	30–40				
	40–50				
	50–60				
	>60				
3. Professional category (reference = category at lower VC%, i.e., nursing stuff)	Nursing staff	0.5 (0.5–0.6)	0.000	2.3 (2.0–2.5)	0.000
	Other healthcare workers	0.6 (0.5–0.6)	0.000		
	Medical staff	18.3 (12.5–26.8)	0.000		
4. Area (reference = area at lower VC%, i.e., surgical area)	servic	0.7 (0.6–0.9)	0.001	1.5 (1.3–1.8)	0.000
	Services	0.9 (0.8–1.1)	0.958		
	Medical	1.3 (1.1–1.5)	0.001		

## Data Availability

The data presented in this study are available on reasonable request from the corresponding author.
